# Serum carotenoids and macular pigment optical density in patients with intestinal resections and healthy subjects: an exploratory study

**DOI:** 10.1017/jns.2017.71

**Published:** 2018-02-05

**Authors:** Jane N. Eriksen, August P. Prahm, Mads Krüger Falk, Eva Arrigoni, Palle B. Jeppesen, Michael Larsen, Lars O. Dragsted

**Affiliations:** 1 Department of Nutrition, Exercise and Sports, University of Copenhagen, Rolighedsvej 30, DK-1958 Frederiksberg C, Denmark; 2 Agroscope, Competence Division Plants and Plant Products, Schloss 1, CH-8820 Wädenswil, Switzerland; 3 Department of Gastroenterology, Rigshospitalet, Blegdamsvej 9, DK-2100 Copenhagen Ø, Denmark; 4 Department of Ophtalmology, Rigshospitalet, Blegdamsvej 9, DK-2100 Copenhagen Ø, Denmark

**Keywords:** Lutein, Zeaxanthin, Macular pigment optical density, Intestinal resection, MPOD, macular pigment optical density

## Abstract

Reduced absorption capacity in patients with intestinal resections (IR) could result in malabsorption of fat-soluble components like carotenoids, which are of clinical interest in relation to visual health. In this case cohort, we investigated the association between IR and serum lutein, zeaxanthin, β-carotene and macular pigment optical density, when compared with healthy controls. Ten patients with IR and twelve healthy controls were included in the study. Baseline characteristics were comparable between groups, except for higher serum TAG (*P* < 0·05) and shorter bowel length (*P* < 0·0001) in the group with IR. Serum lutein, zeaxanthin, β-carotene and macular pigment optical density were >15 % lower in the patient group compared with healthy controls (*P* < 0·05, adjusted for age) and, in the case of serum lutein and zeaxanthin, also for dietary intake of carotenoids. Results suggest that for a test of macular carotenoid supplementation, subjects with a potentially clinically significant carotenoid deficit could be recruited among patients with IR.

Levels of the macular xanthophylls lutein, zeaxanthin and *meso*-zeaxanthin have been associated with slower progression of age-related macular degeneration^(^^1^^)^, potentially through their action in quenching reactive oxygen species, scavenging blue light and thereby protecting the eye from photo-oxidative damage^(^^2^^)^. Carotenoids are not synthesised *de novo* in humans^(^^3^^)^ and we therefore depend on dietary intake and efficacy of absorption for their action. Reduced absorption capacity in the small bowel secondary to surgical resection could thereby put these patients at risk for malabsorption of carotenoids relevant for visual health. In addition, underlying conditions, which influence intestinal mucosa integrity, transport capacity, availability of lipids for carotenoid solubilisation and gastric passage time, are expected to be important parameters for carotenoid absorption capacity^(^^4^^)^. The severity of malabsorption is expected to reflect among other underlying diseases, area of resection, length, and health of the remaining tissue^(^^5^^)^. There is little scientific information, however, about serum levels of non-essential fat-soluble components such as carotenoids and their clinical correlates in patients with altered gut absorption.

The aim of the present study was, through exploratory measures, to investigate levels of serum carotenoids and optical density of the macular pigment in a case cohort of patients with intestinal resection and healthy subjects.

## Subjects and methods

### Subjects and study design

The recruitment methodology is described in detail in the study by JN Eriksen *et al.* (unpublished results) and in the Supplementary material. In brief, we recruited twelve healthy subjects and ten patients with intestinal resections (140–350 cm small intestine left) from a pool of volunteers and from the Department of Gastroenterology of the Rigshospitalet, respectively, to participate in a cross-over study of carotenoid supplementation. The data presented here are from the baseline examination. Subjects were matched by age (±5 years) and sex.

### Ethical approval

The study was approved by The Danish National Committee on Health Research Ethics (study no. H-3-2014-112) and registered at clinicaltrial.gov (study no. NCT02450227).

### Carotenoid dietary intake

Intake of lutein and zeaxanthin was calculated as crude estimates (lutein/zeaxanthin score) and categorised into three groups using a lutein/zeaxanthin screening tool based on intake and relative bioavailability in four food groups (eggs, broccoli, maize, and dark leafy vegetables)^(^^6^^)^.

### Blood sampling, carotenoid extraction and quantification

Methodologies for blood sampling, carotenoid extraction and quantification have been described by Eriksen *et al.*^(^^7^^)^.

### Vision study

Macular pigment optical density (MPOD) was assessed from blue light autofluorescence (BAF) fundus images from both eyes of each subject and analysed using Corel Paint Shop Pro (Corel Corporation). BAF images and optical coherence tomography scans of the retina and structures of the posterior pole of the eye were obtained by scanning laser ophthalmoscope (Heidelberg Spectralis HRA; Heidelberg Engineering GmbH). MPOD was calculated as the density of the fovea relative to the average of two reference areas on the nasal and temporal side of the foveal zone of xanthophyll pigmentation.

### Data analysis

Data processing and statistical analysis were conducted in GraphPad Prism version 6.03 (GraphPad Software Inc., 2013) and R version 3.1.1. (R Core Team, 2013). Continuous variables were checked by the Shapiro–Wilk test for normality. For differences between baseline variables in the two groups, Student's *t* test, the Mann–Whitney non-parametric test and Fisher's exact test were applied. Linear regression models were constructed to test for association between the dependent variables lutein, zeaxanthin, β-carotene and MPOD, and the independent variables bowel length or study group (healthy/intestinal resection). Multiple linear regression models were applied to adjust for confounding variables found to be associated with the dependent variable or if a physiological relationship was found established in the literature. Age was always included in the adjustment.

## Results and discussion

The two study groups, patients with intestinal resection and healthy subjects, were of comparable age and sex distribution (Table 1). The patients with intestinal resection had roughly half the bowel length (140–350 cm, comprising jejunostomy and ileostomy, *P* < 0·0001) and twice the serum TAG level (*P* < 0·05) of the healthy subjects. Patients with intestinal resection were the result of surgical resection for ulcerative colitis (*n* 1), Crohn's disease (*n* 8) and radiation enteropathy (*n* 1). All patients with intestinal resection used oral vitamin supplements and two received intravenous fluid therapy.

**Table 1.** Baseline covariates in the two study groups (Mean values and standard deviations; percentages; medians and ranges; numbers of subjects)

	Intestinal resection (*n* 10)		Healthy (*n* 12)		
	Mean	sd	Mean	sd	*P*
Age (years)	50·2	10·1	49·6	8·2	0·519†
Sex, female (%)	60		67		1·000
BMI (kg/m^2^)	25·2	2·5	24·3	2·5	0·742†
Bowel length (cm)					<0·0001****§
Median	330		650‡		
Range	140–350				
Serum (mmol/l)ǁ					
TAG					0·015*§
Median	1·75		0·72		
Range	0·65–7·07		0·51–1·36		
Cholesterol					0·589§
Median	4·71		4·59		
Range	3·39–6·78		3·65–5·64		
Fasting blood sugar (mmol/l)	5·30	0·25	5·11	0·42	0·136†
Lutein/zeaxanthin score¶ (*n*)ǁ					
1	5		9		0·331††
2	4		2	
3	0		1	

* *P* < 0·05, **** *P* < 0·0001.

† Student's *t* test.

‡ Theoretical value.

§ Mann–Whitney non-parametric test.

ǁ *n* 9 for intestinal resection group.

¶ Score calculated based on intake and relative bioavailability in four food groups.

†† Fisher's exact test.

Differences in serum lutein, zeaxanthin and β-carotene between the two groups are presented in Tables 2 and 3. Circulating levels of carotenoids are expected to reflect carotenoid intake, bioavailability and here also absorption capacity. The three carotenoids lutein, zeaxanthin and β-carotene were higher (*P* < 0·05) in healthy subjects than in patients with intestinal resection (*P* < 0·05, adjusted for age and, in the case of lutein and zeaxanthin, for dietary intake of these two carotenoids). Serum carotenoids and MPOD were also found associated with the continuous variable bowel length (data not presented). The mean (or median) serum concentrations of lutein, zeaxanthin and β-carotene were 0·165, 0·034 and 0·493 µmol/l, respectively, in healthy subjects and 0·115, 0·027 and 0·111, respectively, in patients with intestinal resection (Table 2). Comparable studies in patients with intestinal resection are to our knowledge limited to studies investigating the relationship with underlying disease including ulcerative colitis^(^^8^^)^ and Crohn's disease^(^^9^^)^.

**Table 2.** Baseline characteristics of study participants (Mean values and standard deviations; medians and interquartile ranges)

	Intestinal resection (*n* 10)		Healthy (*n* 12)		
	Mean	sd	Mean	sd	*P*
Serum carotenoids (μmol/l)					
Lutein	0·115	0·078	0·165	0·053	0·092†
Zeaxanthin					0·099‡
Median	0·027		0·034		
Interquartile range	0·001–0·038		0·027–0·044		
β-Carotene					0·022*‡
Median	0·111		0·493		
Interquartile range	0·001–0·420		0·0395–0·624		
Macular pigment optical density	0·506	0·113	0·606	0·098	0·043*†

* *P* < 0·05.

† Student's *t* test.

‡ Mann–Whitney test.

**Table 3.** Cross-sectional associations for difference in serum carotenoids (μmol/l) and macular pigment optical density between short-bowel patients and age (± 5 years)- and sex-matched controls (β-Coefficients and standard errors)

	Unadjusted			Adjusted		
	β	se	*P*	β	se	*P*
Serum lutein (μmol/l)						
Group difference	0·0502	0·0283	0·0923	0·0618	0·0267	0·0341*
Full age adjustments†				−0·0035	0·0015	0·0303*
Lutein/zeaxanthin score adjustments				–	–	–
2				0·0537	0·0293	0·0857
3				0·0226	0·0608	0·7156
Serum zeaxanthin (μmol/l)						
Group difference	0·0112	0·0066	0·1069	0·0138	0·0061	0·0382*
Full age adjustments				−0·0008	0·0003	0·0320*
Lutein/zeaxanthin score				–	–	–
2				0·0126	0·0067	0·0788
3				0·0069	0·0139	0·6291
Serum β-carotene (μmol/l)						
Group difference	0·1924	0·0882	0·0421*	0·1879	0·0789	0·0284*
Full age adjustments				−0·0106	0·0044	0·0268*
Macular pigment optical density						
Group difference	0·0999	0·0001	0·0125*	0·1029	0·0395	0·0181*
Full age adjustments				−0·0064	0·0023	0·0188*

* *P* < 0·05.

† Age-matching was performed in a broad interval and these adjustments correct for residual age differences arising from unequal group size.

Serum carotenoids may affect visual health in several ways. β-Carotene is associated with visual health through its role as a pro-vitamin A carotenoid, whereas lutein and zeaxanthin are found as pigments in the macula lutea. MPOD levels were significantly lower in patients with intestinal resection compared with healthy controls (Tables 2 and 3; *P* < 0·05). Comparable studies have to our knowledge not been published at the present time and evidence on visual complications secondary to malabsorption of vitamin A and carotenoids in related patient groups is limited to a few case studies^(^^10^^,^^11^^)^. Supporting evidence can, however, be found in publications investigating serum carotenoid levels, MPOD and objective measures of visual function in cystic fibrosis patients. It has been suggested that absorption of carotenoids in this patient group could be hampered by pancreatic insufficiency not fully substituted by enzyme replacement therapy. Two small studies with nine and ten cystic fibrosis patients showed lower serum carotenoids and lower MPOD, but did not find a significant effect on visual acuity^(^^12^^,^^13^^)^. Evidence is, however, still lacking for the effect of long-term decrease in macula pigments and the risk of later visual complications.

The present study is limited in sample size and inter-correlation between absorption capacity, serum levels, and the functional markers of vision, MPOD, makes analyses challenging. Future studies should focus on the physiological effect of reduced circulating carotenoid levels on MPOD in patient groups with malabsorption, potentially with additional measurements of other fat-soluble vitamins.

### Conclusion

The present study found lower serum carotenoids and lower macular pigment optical densities in patients with intestinal resection than in healthy subjects. While there is no certain knowledge about the effects of having low serum carotenoid levels, there are important indications that low retinal carotenoid levels may have a deleterious effect on retinal health. Our findings suggest that patients with intestinal resection or even short bowel disease should be examined, in a scientific context, for signs of poor retinal health. Our results should be validated in larger studies.

## Supplementary material

For supplementary material accompanying this paper visit https://doi.org/10.1017/jns.2017.71. click here to view supplementary material
