# Supplementary material for: Serum carotenoids and macular pigment optical density in patients with intestinal resections and healthy subjects: an exploratory study
Source: J Nutr Sci. 2018 Feb 5;7:e8. doi: 10.1017/jns.2017.71 (PMC5799610; doi:10.1017/jns.2017.71)
Supplement: Supplementary file 1 [file S2048679017000714sup001.doc]

**Supplementary material**

**Serum carotenoids and macular pigment optical density in patients with intestinal resections and healthy subjects: an exploratory study**

^a,b^Jane N. Eriksen^1^, ^c^August P. Prahm, ^d^Mads Krüger Falk, ^b^Eva Arrigoni, ^c^Palle B. Jeppesen, ^d^Michael Larsen,  ^a^Lars O. Dragsted

^a^Department of Nutrition, Exercise and Sports, University of Copenhagen, Rolighedsvej 30, DK-1958 Frederiksberg C, Denmark

^b^Agroscope, Competence Division Plants and Plant Products, Schloss 1, CH-8820 Wädenswil, Switzerland

^c^Department of Gastroenterology, Rigshospitalet, Blegdamsvej 9, DK-2100 Copenhagen Ø, Denmark.

^d^Department of Ophtalmology, Rigshospitalet, Blegdamsvej 9, DK-2100 Copenhagen Ø, Denmark.

Corresponding author^1^: Jane N. Eriksen, Tel: +45 35331084, e-mail: [j-n-e@nexs.ku.dk](mailto:j-n-e@nexs.ku.dk)

**Supplementary material: Study design and recruitment methodology**

The presented data are part of a larger intervention trial called **“Carotenoid absorption and metabolism from green-leafy vegetable matrices – a explorative study in short-bowel patients and healthy controls – The “Popeye-study**”. The data presented here are from the baseline examination. The study was approved by The Danish National Committee on Health Research Ethics (study no. H-3-2014-112) and registered at clinicaltrial.gov (study no. NCT02450227).

# Study hypothesis.

# "Patients with gastrointestinal diseases and reduced absorption capacity secondary to intestinal surgery are at risk of lower absorption of carotenoids from green leafy vegetables, and thereby for development of AMD".

**Overall objectives.**

1. To validate an *in vitro* digestion method as a screening tool for carotenoid bio-accessibility from green-leafy vegetables matrices and, based on this, a means to esti­mate the bioavailability potential in humans.
2. To investigate baseline levels, absorption and metabolism of carotenoids from green-leafy vegetables in patients with surgical altered gut absorption, hereunder to compare levels found in this patient group with levels in healthy individuals.

**Details on subjects and methods**

***Subjects.*** Subjects were recruited from the city of Copenhagen and from the Gastroenterology Department at Rigshospitalet, Denmark into two groups: healthy (n = 12) and short bowel subjects (n = 10), respectively (Supplementary Fig. S1). Subjects were matched based on age (± 5 years) and sex.

Eligibility criteria for both groups included

- Adults (18-80 years of age)
- BMI between 18.9-28.0
- Normal fasting blood glucose levels (3.9 - 5.6 mmol/L).

For short bowel subjects additional inclusion criteria comprised

- Period after surgery > 1 year and stability in underlying disease.
- Information on short bowel/ileostomy patients was obtained regarding type and doses of vitamin and other type of supplementation used, time since operation, type of disease before surgery, amount (length), and area of dissection.

Exclusion criteria included

- Bowel diseases (for healthy controls), systemic infections, psychiatric disorders and metabolic disorders (including diabetes), as well as any clinical condition or other condition that, according to the investigator's assessment, makes the person unsuitable for participation in the trial.
- Intake of vitamin pills or other supplements (3 months before the start of the trial and throughout the trial) (healthy controls). For the patient group, subject are only included with stable intake of dietary supplements over the last 3 months.
- Medication interfering with fat-related absorption, systemic treatment with glycocorticoids, or anti-coagulant medication (Marevan)
- Intake of carotenoid containing supplements (only for healthy subjects)
- Alcohol consumption in excess of the recommended (7/14 drinks weekly for females/males)
- Smooking or former drug addition.
- People with known allergies towards spinach or other green-leafy vegetables
- Patients with diagnosed short-bowel-syndrome (SBS).
- Delivery of a larger amount of blood (example: in connection with donation or other scientific trials) 3 months before the start of the trial and until its completion.
- Women who are breastfeeding, pregnant or planning pregnancy
- Diagnosed Age-related macular degeneracy (AMD) of a severity greater than that of One or more drills larger than 125 microns in diameter for at least one eye.
- Cataract, macular disease or other eye diseases that prevent xanthophyll imaging.


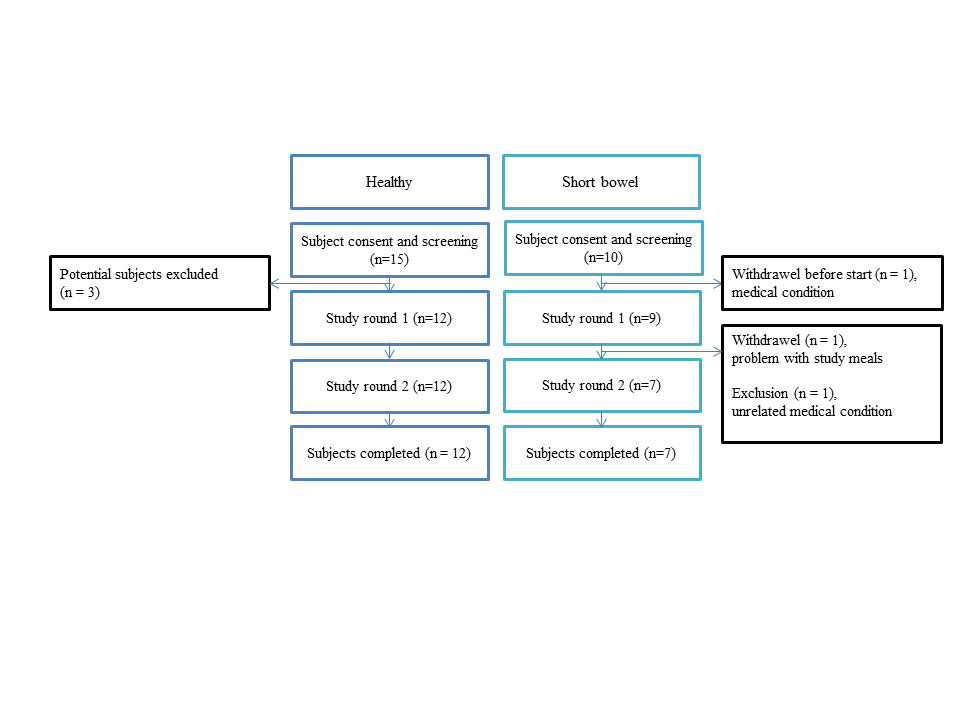


Supplementary Fig. S1. Consort flow diagram.

***Details on study design.*** At screening, subjects were screened according to specified inclusion and exclusion criteria. Subsequently, participants were subjected to an eye examination. For both trial groups, routine clinical determination of visual strength, color vision and contrast perspective, and routine fundus photography were performed in different illumination colors and optical coherence tomography.

Subjects were afterwards randomized to two study periods with spinach preparations, which were expected to differ in carotenoid bio-accessibility based on previous findings ^(1)^. Each study period was sub-divided into three parts: run-in period (-48 - 0 hours), test day (0 – 24 hours), and follow-up period (24 hours – 2 weeks) (Supplementary Fig. S2). In the run-in periods, subjects were asked to avoid foods rich in carotenoids according to a provided list. Apart from intervention foods, the participants were asked to keep to their habitual diet during the follow-up period. The two study periods were separated by a 2-weeks wash-out period.


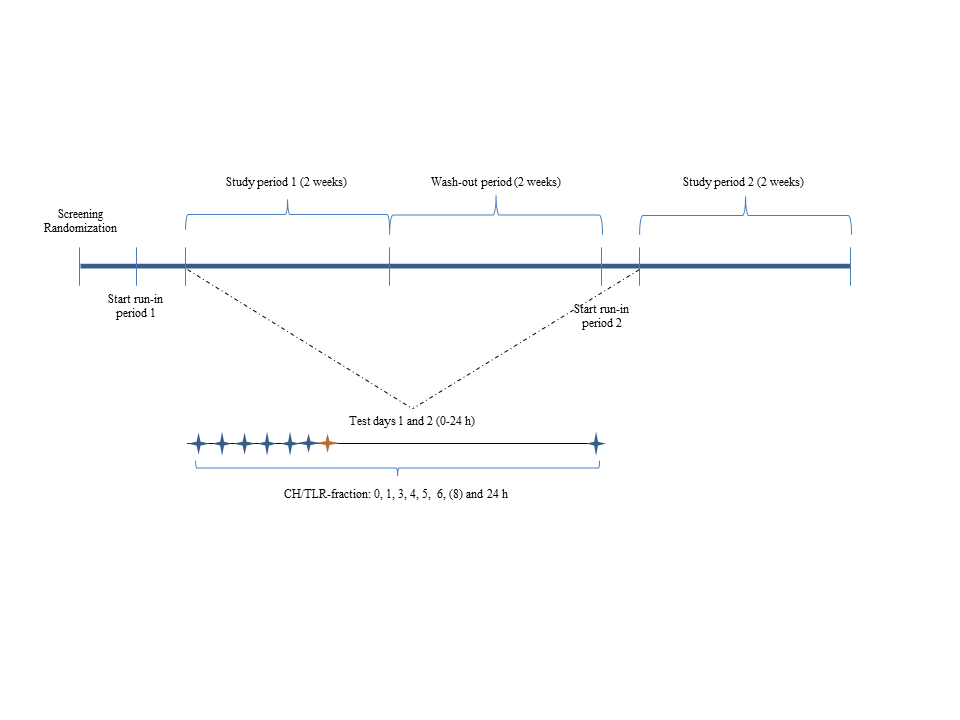


Supplementary Fig. S2. Study design.

On the test day, subjects were asked to come fasting since the intake of a standardized low carotenoid meal the evening before. The test meal was served as breakfast after collection of baseline samples.

***Details on habitual intake of carotenoids from food.*** Habitual carotenoid intake from foods was assessed by semi-quantitative food frequency questionnaire. Intake of lutein and zeaxanthin was calculated as crude estimates and categorized into three groups using a lutein/zeaxanthin-screening tool based on intake and relative bioavailability in four food groups (eggs, broccoli, corn, and dark leafy vegetables)^(2)^.

***Details on test meals.*** Test meals were composed of 178 g of whole leaf spinach (lutein: 9.9 mg per portion; β-carotene: 8.7 mg per portion) and 200 g of minced spinach (lutein: 10.8 mg per portion; β-carotene: 9.0 mg per portion) containing 7.1 and 8.0 g of rapeseed oil, respectively. The test meals were served in the morning with 400 mL water. Additionally, standardized low carotenoid diets were supplied to the subjects the evening before the test day, at lunch and dinner on the test day. The lunch and dinner meals contained 2500 kJ and 3000 kJ of total energy, respectively. For both meals the macronutrient distribution was: 15 E% protein, 30 E% fat and 55 E% carbohydrates. The meals were designed to contain a minimum amount of carotenoids < 500 µg/meal. Standardized snacks low in carotenoids were additionally supplied in the afternoon and evening on the test day. Test meals prepared according to randomization were provided to participants to be consumed every second day in the two follow-up period.

***Details on in vitro digestion.*** Carotenoid liberation and *in vitro* accessibility from the identical spinach preparations as provided to volunteers were evaluated by *in vitro* digestion. The methodology was as previously described for screening purposes ^(1; 3)^. In short, 5 g of spinach + 200 mg of rapeseed oil were digested in a 3-step static *in vitro* digestion method in triplicate at four different occasions over a 6 months period. Carotenoid liberation was determined in the aqueous phase of digesta after centrifugation, whereas *in vitro* accessibility was assessed in micelles recovered from the aqueous phase after filtration through 0.22 μm. For details see Eriksen et al. ^(1)^ and Eriksen et al.^(3)^

***Detail on blood sampling and analysis.*** Blood samples for plasma were drawn at 0 hours and at 1, 3, 4, 5, 6, 8 (only for subjects in the short bowel group), and 24 hours postprandially. Fasting serum samples were collected at baseline, 8 and 15 days in each intervention period.

TLR/CH fractions were obtained from EDTA-plasma by ultracentrifugation according to the methodology by Eriksen et al.^(4)^. TLR/CH fraction and serum samples were analysed for carotenoids (lutein, zeaxanthin, beta-carotene, alpha-carotene and lycopene), triglyceride and cholesterol at all-time points. Triglyceride and cholesterol analyses were performed on an ABX Pentra 400 (Horiba Medical, Brøndby, Denmark).

***Details on carotenoid extraction and quantification.*** Carotenoid content in test meals, digesta and blood samples were extracted as described previously ^(1; 4)^ and analysed by UHPLC coupled to a DAD detector ^(4)^. In brief, extraction was conducted in triplicate with methanol:acetone (1:1, v/v, 0.01% BHT) from test meals (1.5 g:100 mL) and from digesta (5 mL:5 mL)*.* Heptane with 0.01% BHT was used to extract carotenoids from both serum (0.4 mL:0.5 mL) and TLR/CH fractions (2.4 mL:1.5 mL), respectively. Carotenoids were quantified against external calibration curves with standards obtained from Carotenature (Lupsingen, Switzerland).

**References**

1. Eriksen JN, Luu AY, Dragsted LO *et al.* (2016) In vitro liberation of carotenoids from spinach and Asia salads after different domestic kitchen procedures. *Food Chemistry* **203**, 23-27.

2. Moran R, Johnson EJ, Stack E *et al.* (2014) The Relationship Between Dietary Intake of Lutein and Zeaxanthin and Their Concentration in Serum: Introduction of a Novel Carotenoid Dietary Screener. In *International Carotenoid Symposium* Park City, Utah.

3. Eriksen JN, Luu AY, Dragsted LO *et al.* (2017) Adaption of an In Vitro Digestion Method to Screen Carotenoid Liberation and In Vitro Accessibility from Differently Processed Spinach Preparations. *Food Chemistry* **224**, 407-413.

4. Eriksen JN, Madsen P, Dragsted LO *et al.* (2016) Optimized, fast through-put UHPLC-DAD based method for carotenoid quantification in green-leafy vegetables, serum, chylomicrons and faeces.  *Journal of Agricultural and Food Chemistry* **65**, 973-980.
